# Identification of a novel RhlI/R-PrrH-LasI/Phzc/PhzD signalling cascade and its implication in *P. aeruginosa* virulence

**DOI:** 10.1080/22221751.2019.1687262

**Published:** 2019-11-12

**Authors:** Yang Lu, Honglin Li, Jieying Pu, Qian Xiao, Chanjing Zhao, Yimei Cai, Yuyang Liu, Lina Wang, Youqiang Li, Bin Huang, Jianming Zeng, Cha Chen

**Affiliations:** a Department of Laboratory Medicine, The Second Affiliated Hospital of Guangzhou University of Chinese Medicine, Guangzhou, P. R. People’s Republic of China; b The Second Clinical College of Guangzhou University of Chinese Medicine, Guangzhou, P. R. People’s Republic of China; c Department of Laboratory Medicine, The First Affiliated Hospital of Sun Yat-sen University, Guangzhou, P. R. People’s Republic of China

**Keywords:** PrrH, sRNA, quorum sensing, P. aeruginosa, virulence

## Abstract

Small regulatory RNAs (sRNAs) act as key regulators in many bacterial signalling cascades. However, in *P. aeruginosa*, the sRNAs involved in quorum sensing (QS) regulation and their function are still largely unknown. Here, we explored how the *prrH* locus sRNA influences *P. aeruginosa* virulence in the context of the QS regulatory network. First, gain- and loss-of-function studies showed that PrrH affects pyocyanin, elastase and rhamnolipid production; biofilm formation; and swimming and swarming motility and impaired the viability of *P. aeruginosa* in human whole blood. Next, our investigation disclosed that LasI and PhzC/D were directly repressed by PrrH. In addition, RhlI, the key member of the *rhl* QS system, diminished the expression of PrrH and enhanced the expression of downstream genes. Bioinformatics analysis found two binding sites of RhlR, the transcription factor of the *rhl* system, on the promoter region of *prrH*. Further β-galactosidase reporter and qPCR assays confirmed that PrrH was transcriptionally repressed by RhlR. Collectively, our data identified a novel RhlI/R-PrrH-LasI/PhzC/PhzD regulatory circuitry that may contribute to *P. aeruginosa* pathogenesis. Our findings indicate that PrrH is a quorum regulatory RNA (Qrr) in *P. aeruginosa* and provide new insight into PrrH’s function.

## Introduction

The opportunistic pathogen *Pseudomonas aeruginosa* (*P. aeruginosa*) is the cause of a variety of human infections, including acute infections in patients who are injured or severely burned and chronic lung infections in individuals with cystic fibrosis [1]. *P. aeruginosa* pathogenicity is due to mainly a large amount of cell-associated and extracellular virulence factors, many of which are regulated by two acyl-homoserine lactone (AHL) quorum sensing (QS) systems, LasI-LasR (*las* system) and RhlI-RhlR (*rhl* system) [2]. In the *las* system, the *lasI* gene product directs the formation of the diffusible extracellular signal N-(3-oxododecanoyl)-L-HSL (3-oxo-C12-HSL), which interacts with LasR to activate a number of virulence factors, including the LasA and LasB elastases, exotoxin A, and alkaline protease. In an analogous manner, the *rhlI* and *rhlR* genes encode for an N-butanoyl-L-homoserine lactone (C4-HSL) synthase and receptor, respectively. The downstream virulence genes of the *rhl* system include those encoding pyocyanin, rhamnolipids and the type III secretion system [3,4]. In commonly studied *P. aeruginosa* strains, *las* and *rhl* systems are hierarchically connected, and both *rhlR* and *rhlI* are positively regulated by the *las* system [5]. However, recent research has shown that once LasR mutants emerge among populations capable of C4-HSL production, they can rapidly evolve an active LasR-independent *rhl* QS system [2,6]. In addition, these two AHL QS systems interact with a non-AHL signalling system called the *Pseudomonas* quinolone signal (PQS) system, which is regulated by mainly the *pqs* operon [7].

Small regulatory RNAs (sRNAs) are central regulators of gene expression in bacteria and post-transcriptionally control target genes by limited base pairing with their mRNAs, thus negatively or positively affecting the transcript stability and/or the translation rate [8,9]. In recent years, increasing studies have shown that sRNAs regulate a wide variety of genes that encode for proteins involved in many cellular processes and pathogenesis, including QS and virulence activities [10–12]. In *P. aeruginosa*, ReaL is negatively regulated by the *las* system and enhances the synthesis of PQS by targeting PqsC [13]. In addition, PhrS and PrrF modulate PQS by regulating PqsR and AntR, respectively [14–17]. However, the number of sRNAs that have been implicated in the regulation of QS, especially the *las* and *rhl* system, is limited. An extensive investigation of quorum regulatory RNAs (Qrrs) [18] will help to disclose the elaborate regulatory network of *P. aeruginosa* virulence.

PrrH is characterized as a haem-responsive sRNA encoded by the *prrF* locus. Transcription of PrrH initiates at the 5’ end of *prrF1*, proceeds through the *prrF1* terminator and *prrF1*-*prrF2* intergenic sequence, and terminates at the 3’ end of the *prrF2* gene. The widely known function of this sRNA family is its participation in iron homeostasis, homologous to the RyhB RNAs encoded by *Escherichia coli*, *Shigella flexneri*, *Shigella dysenteriae*, and *Vibrio cholerae* [19,20]. In this study, we showed that perturbation of PrrH levels affected many processes that are known to be influenced by QS and regulated the virulence of *P. aeruginosa*. Further evidence demonstrated that the LasI and *phzA-G* operons were direct targets of PrrH. More importantly, we also disclosed that the virulence inhibition function of PrrH was under the control of the *rhl* system. Our findings highlight the importance of PrrH in *P. aeruginosa* pathogenesis and implicate PrrH as a potential therapeutic target for *P. aeruginosa*.

## Results

PrrH influences *P. aeruginosa* virulence and bloodstream infection ability *in vitro*.

**Figure 1.**
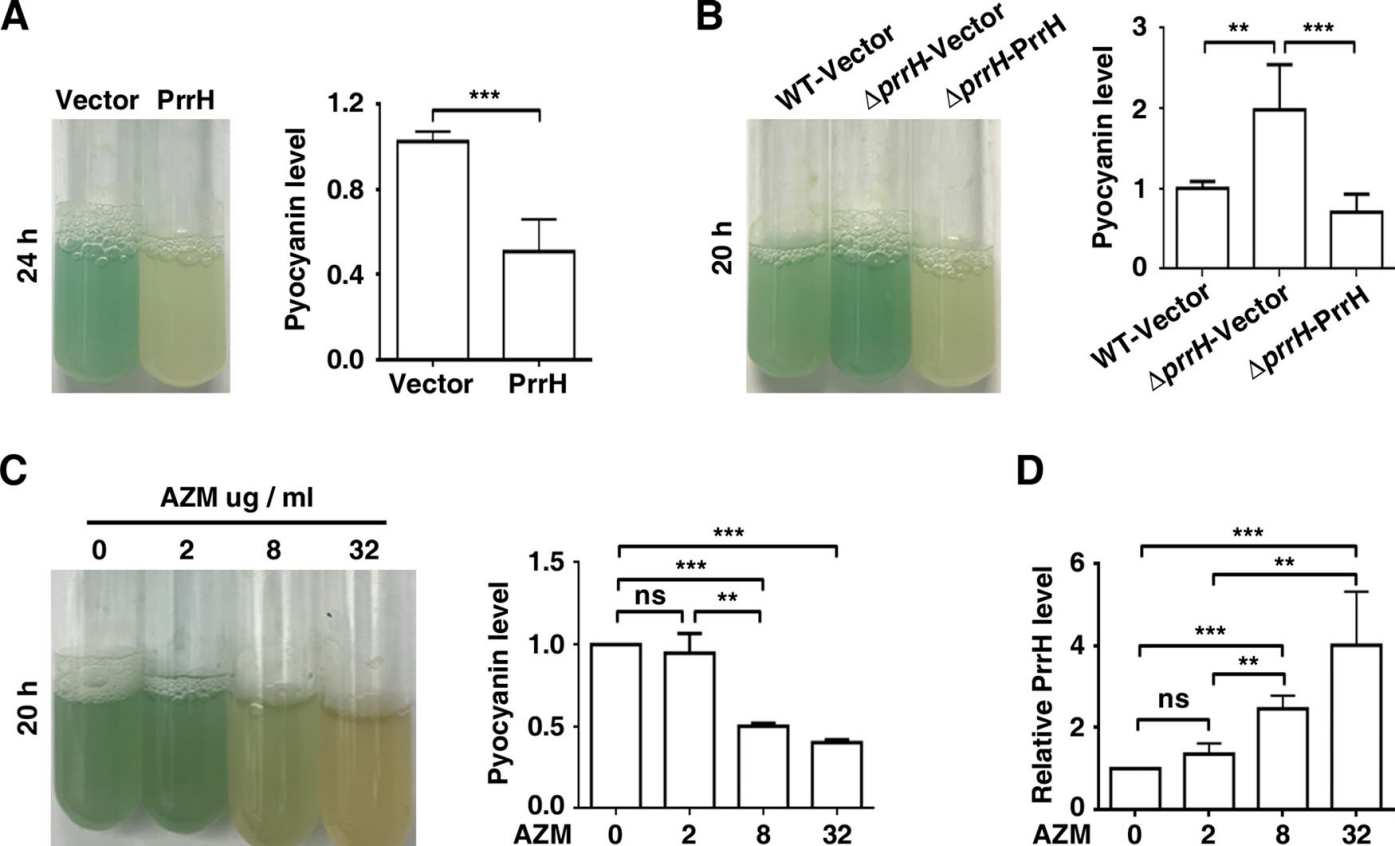
PrrH suppresses pyocyanin synthesis of *P. aeruginosa*. (A) PrrH overexpression inhibited pyocyanin production. PAO1 carrying the pROp200 vector (Vector) or pROp200-*prrH* (PrrH) was cultured in LB for 24 h, and pyocyanin in the supernatant was measured. (B) Deficiency in *prrH* resulted in an increase in pyocyanin, and complementation with *prrH* abolished pyocyanin production. PAO1 and PAO1*ΔprrH* carrying pROp200 (WT/Vector, *ΔprrH*/Vector) or pROp200-*prrH* (*ΔprrH*/PrrH) were cultured in LB for 20 h, and pyocyanin in the supernatant was measured. (C, D) Pyocyanin production was negatively correlated with PrrH expression under the treatment of AZM. PAO1 was treated with 2, 8 or 32 μg/mL of AZM for 24 h, followed by pyocyanin measurement (C) or real-time PCR analysis (D). The *rpoD* gene was used as an internal control. Values are the mean ± SD of at least three independent experiments. ns, not significant; **, *P* < 0.01; ***, *P* < 0.001.

To assess the function of PrrH, we first constructed *prrH* gene-deficient and overexpression strains in PAO1 (*ΔprrH*, WT/PrrH, respectively), as well as a *prrH*-overexpression strain in the *ΔprrH* mutation background (*ΔprrH/*PrrH). The expression levels of the PrrH in all these strains were confirmed by RT-qPCR (Figure S1). Compared to the wild-type strain, the strain with overexpression of *prrH* had slightly repressed growth, and deficiency in *prrH* barely affected growth (Figure S2). However, it was notably that overexpression of *prrH* inhibited the production of pyocyanin (Figure 1A), while deficiency in *prrH* in the genome resulted in an increase in pyocyanin, which was attenuated by introduction of a *prrH*-overexpression plasmid (Figure 1B). Furthermore, as azithromycin (AZM) has been shown to potentially inhibit QS signal molecules and attenuate pyocyanin synthesis by *P. aeruginosa* [21,22], we found that when *P. aeruginosa* was exposed to 2, 8, and 32 μg/ml AZM (1/64th, 1/16th, and 1/4th of the MIC), pyocyanin synthesis was decreased in a dose-dependent manner (Figure 1C), accompanied with enhanced expression of PrrH, also in a dose-dependent manner (Figure 1D). These data suggest that PrrH negatively regulates pyocyanin synthesis in *P. aeruginosa*.

**Figure 2.**
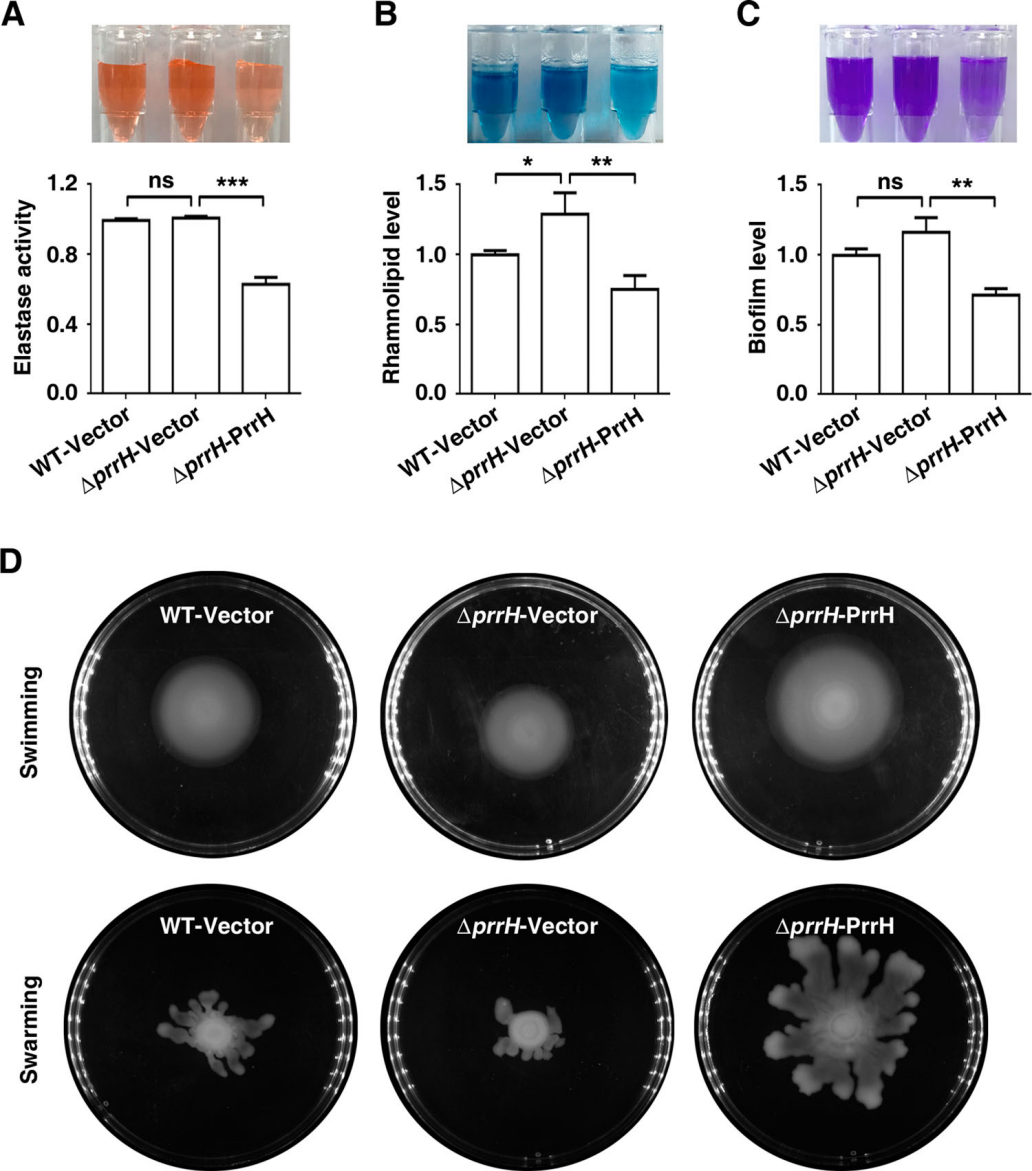
PrrH influences elastase and rhamnolipid production, biofilm formation, swimming and swarming motility of *P. aeruginosa*. (A) PrrH overexpression inhibited elastase production. PAO1 carrying pROp200 (WT/Vector), PAO1*ΔprrH* carrying pROp200 or pROp200-*prrH* (*ΔprrH*/Vector, *ΔprrH*/PrrH) were cultured in LB for 8 h, and the activity of elastase in supernatant was determined. (B) PrrH overexpression inhibited rhamnolipid synthesis. The indicated PAO1 strains were cultured in M9 minimal salts medium for 8 h at 37 °C, and rhamnolipid in the supernatant was measured. (C) PrrH inhibited biofilm formation. The indicated PAO1 strains were cultured in LB in the 12-well plates for 24 h at 37°C. The biofilm was quantified by measuring solubilized crystal violet staining biofilm cells at OD600. (D) PrrH positively regulated swimming and swarming motility. 5μl cultures of the indicated strains were spotted onto the swarming or swimming medium and incubated at 37°C for 16 h. Values are the mean ± SD of at least three independent experiments. ns, not significant; **, *P *< 0.01; ***, *P *< 0.001.

To further measure the effects of PrrH on the other QS-linked virulence traits, we next analysed elastase and rhamnolipid production, biofilm formation, and swimming and swarming motility in conditions of *prrH* overexpression or deletion. As shown in Figure 2A, B and C, deletion of *prrH* showed no or little promotion of elastase production, rhamnolipid synthesis and biofilm formation, while PrrH overexpression significantly inhibited the above phenotypes. In contrast, swimming and swarming were slightly repressed by deletion of *prrH* and obviously enhanced by PrrH overexpression (Figure 2D). PrrH appeared to have opposite effects on swimming and swarming compared to those on biofilm formation. This result was consisted with the observation that swarming motility and biofilm formation have an inverse relationship in *P. aeruginosa* strains [13]. Overall, these results indicated that PrrH plays a negative regulatory role in elastase production, rhamnolipid synthesis and biofilm formation, while its role in swimming and swarming motility is positive.

**Figure 3.**
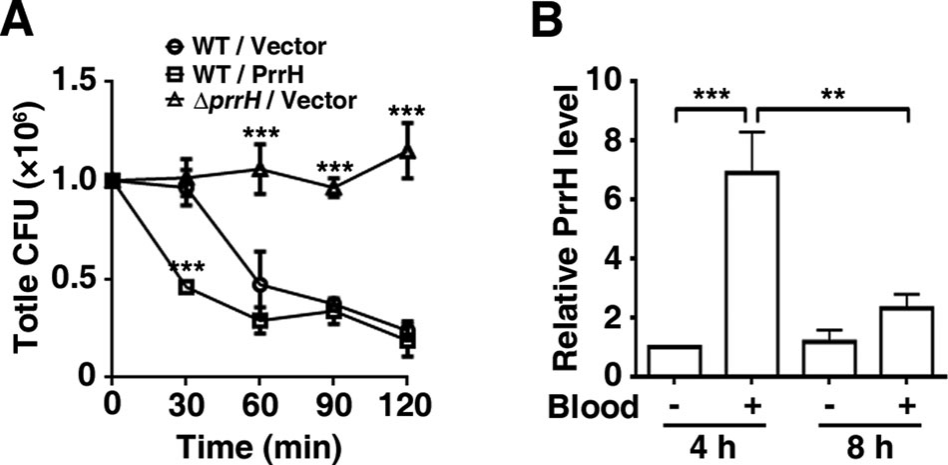
PrrH inhibits *P. aeruginosa* bacteraemia *in vitro*. (A) PrrH repressed *P. aeruginosa* survival in whole blood. Here, 1.0 × 10^6^ CFU of wild-type PAO1 (WT/Vector) and *prrH*-overexpression (WT/PrrH) or *prrH*-deficient (*ΔprrH*/Vector) strains were cultured in whole blood for 30, 60, 90 and 120 min. ***P <0.001, compared with the wild-type strain. (B) The expression of PrrH was enhanced by whole blood. PAO1 was cultured in LB with or without 10% whole blood (indicated as ‘‘+’’ and ‘‘-’’, respectively) for 4 or 8 h, followed by real-time PCR analysis. The *rpoD* gene was used as an internal control. Values are the mean ± SD of at least three independent experiments; **, *P* < 0.01; ***, *P* < 0.001.

Since virulence may be a contributing factor for *P. aeruginosa* bacteraemia [23], we then examined the effect of PrrH on the ability of *P. aeruginosa* to survive in a bloodstream infection by transferring growing bacteria to whole blood to mimic their transition from an extravascular site into the bloodstream [24,25]. As shown in Figure 3A, within 30 min after incubation in whole blood from human volunteers, the count of the *prrH*-overexpression strain was dramatically reduced, while that of the wild-type or *prrH*-deficient strain hardly changed. At 60, 90 and 120 min, more than half of both the wild-type and *prrH*-overexpression PAO1 was killed compared with the initial inoculation quantity. However, it was intriguing that the number of the *ΔprrH* strain was almost constant throughout the incubation. To further investigate the role of PrrH and in *P. aeruginosa* bacteraemia, we tested the expression level of PrrH after whole blood incubation. Allowing for the great differences in RNA quality between these two cultures from Luria–Bertani (LB) medium and whole blood, we reduced the application number in whole blood to 10% and extended the incubation time. The result showed that the expression of *prrH* was enhanced at 4 h and faded at 8 h (Figure 3B). The upregulation of PrrH was coincident with the death of PAO1, which might be explained by a disinfection effect of blood in the early stage, while the reduction might be a result of bacterial adaption to blood.

Collectively, these data suggest that PrrH plays a crucial role in impairing the virulence and bacteraemia of PAO1.

### PrrH directly regulates LasI, PhzC and PhzD expression

**Figure 4.**
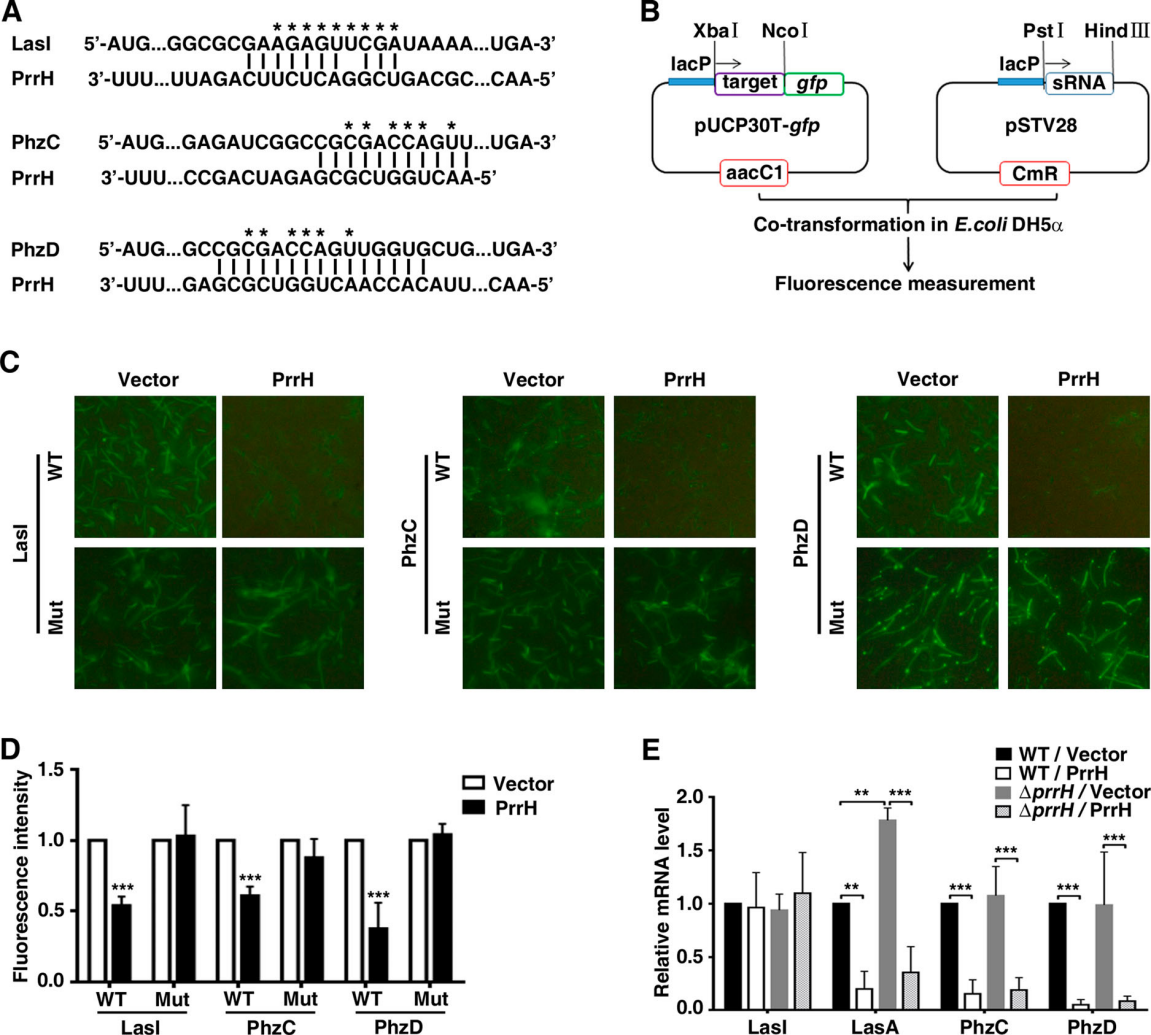
LasI, PhzC and PhzD are direct targets of PrrH. (A) PrrH and its putative binding sequence in the CDS of LasI, PhzC and PhzD. Asterisks denote the mutant PrrH-binding site that was generated as the complementary sequence. (B) Principles of the *in vivo* investigation of direct interactions between sRNAs and their targets in the *E. coli* DH5α strain*.* (C, D) PrrH suppressed the intensity of GFP through its binding sequences at the CDS of target genes. DH5α strains were co-transformed with pROp200 (Vector) or pROp200-*prrH* (PrrH) and a GFP reporter containing a wild-type or mutant CDS of target genes (indicated as WT or Mut); GFP was observed by fluorescence microscopy (C), and the intensity was measured by a Tecan Infinity M1000Pro Reader and expressed in AU as F485/535/Abs595 (D). ***P < 0.001, compared with pROp200-transformants. (E) Effect of *prrH* overexpression or deficiency on the endogenous level of LasI, LasA, PhzC and PhzD. PAO1 and PAO1*ΔprrH* carrying pROp200 (WT/Vector, *ΔprrH*/Vector) or pROp200-*prrH* (WT/PrrH, *ΔprrH*/PrrH) were cultured in LB for 6 h before qPCR analysis, and the *rpoD* gene was used as an internal control. Values are the mean ± SD of at least three independent experiments; **, *P* < 0.01; ***, *P* < 0.001.

Next, we explored the molecular mechanisms responsible for the functions of PrrH that were observed above. Potential target genes of PrrH were first predicted using the publicly available database IntaRNA. LasI, PhzC and PhzD were chosen for further analysis (Figure 4A) because LasI was the key member of a QS system, while phenazine operons, *phzA-G*, played a vital role in pyocyanin synthesis [26]. To verify whether LasI, PhzC and PhzD are direct targets of PrrH, a green fluorescent protein (GFP) reporter system was first constructed (Figure 4B). Next, analysis showed that co-expression of PrrH significantly inhibited the intensity of GFP that carried a wild-type but not mutant base-pairing site of LasI, PhzC or PhzD (Figure 4C and D), indicating that PrrH may suppress gene expression through its base-pairing sequence at the CDS of a target gene. Further investigation showed that PrrH can also diminish endogenous expression of both PhzC and PhzD (Figure 4E), which may be attributed to degradation caused by sRNA. For LasI, the mRNA level was hardly affected by PrrH, however, the mRNA level of *lasA,* a downstream virulence gene of LasI, was significantly decreased upon PrrH overexpression, suggesting that PrrH regulates LasI expression at the translational level (Figure 4E).

Taken together, these results implicate that PrrH may negatively regulate lasI, PhzC and PhzD expression by directly targeting the CDS of their mRNAs.

### Regulation of PrrH transcription by RhlI/R

**Figure 5.**
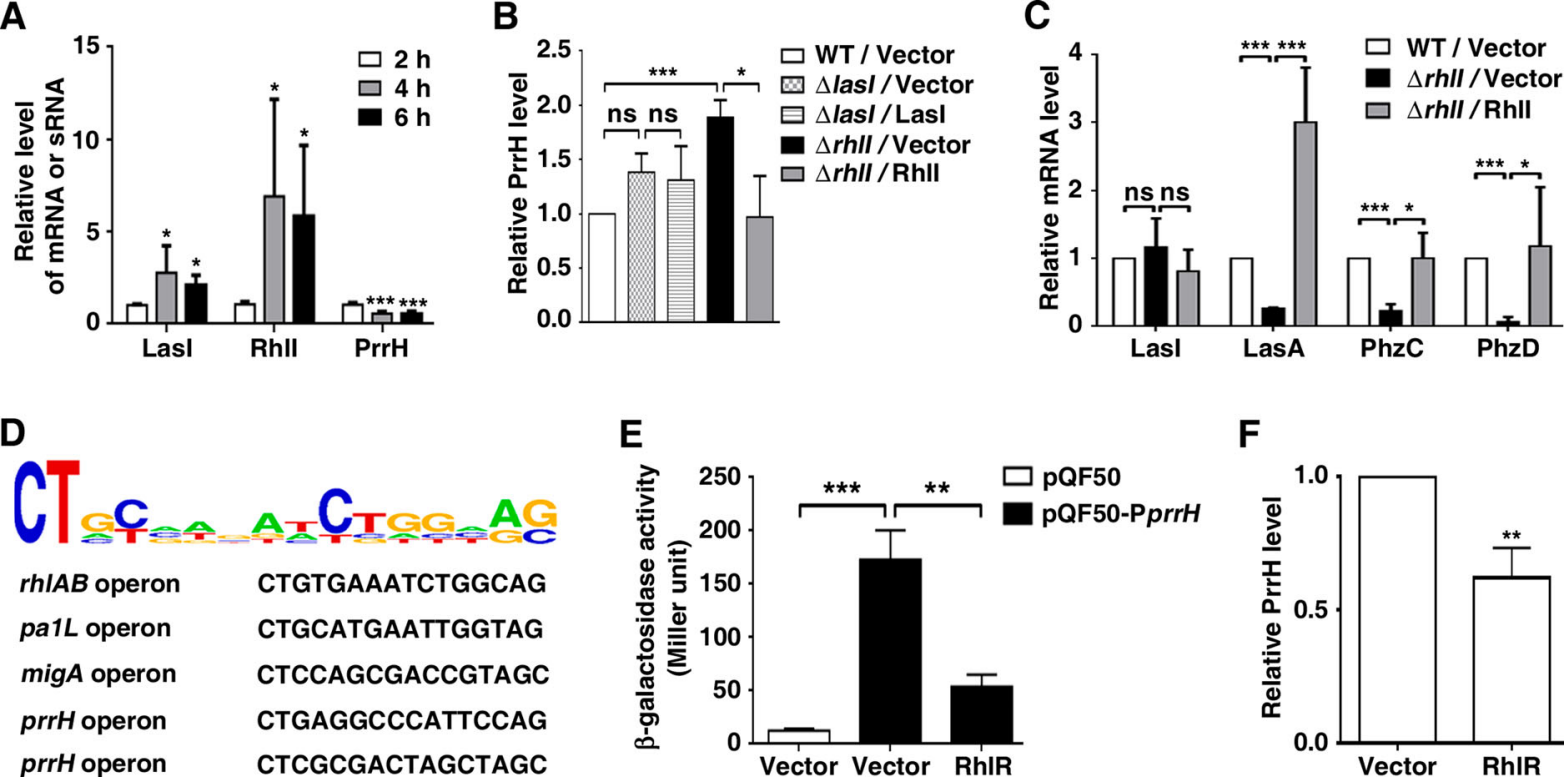
PrrH is negatively regulated by RhlI/R. (A) The expression of PrrH was negatively related to LasI and RhlI. PAO1 in the exponential phase was collected and re-cultured in LB for 2, 4 or 6 h, followed by qPCR analysis. (B) RhlI repressed the expression of PrrH. (C) The target genes of PrrH were regulated by RhlI. (B, C) PAO1 (WT) and the *lasI-* or *rhlI-*deficient strains (*ΔlasI, ΔrhlI*) carrying pROp200 (Vector), pROp200-*lasI* (LasI) or pROp200-*rhlI* (RhlI) were cultured in LB for 6 h before qPCR analysis, and the *rpoD* gene was used as an internal control. (D) Diagram of RhlR-binding sites in the promoter of its putative target genes. (E) Overexpression of RhlR decreased *prrH* promoter activity. The BW25113 strains carrying the reporter pQF50 or pQF50-P*prrH* combined with pROp200 or pROp200-*rhlR* were grown to mid-log phase and subjected to a β-galactosidase activity assay. (F) RhlR repressed the expression of PrrH. The indicated PAO1 strains were cultured for 6 h, followed by real-time PCR analysis. Values are the mean ± SD of at least three independent experiments. ns, not significant; *, *P* < 0.05; **, *P* < 0.01; ***, *P* < 0.001.

Considering that the synthesis of virulence factors is primarily under the control of QS [2], we inferred that PrrH may be transcribed by QS-related genes. Since the activation of QS depends on the density of bacteria, we tested the expression levels of *lasI*, *rhlI* and *prrH* in PAO1 during the early exponential phase (t = 2 h), the mid-exponential phase (t = 4 h) and the early stationary phase (t = 6 h) in a corresponding culture model (Figure S3). As shown in Figure 5A, the mRNA of LasI and RhlI gradually rose from 2 h to 4 h and remained stable at 6 h; it’s important that PrrH displayed an inverse trend of expression over time. Moreover, the expression of PrrH was also negatively related to LasI and RhlI in the whole blood culture model (Figure S4). Therefore, we evaluated the role of LasI and RhlI in PrrH transcription. The results showed that deletion or overexpression of *lasI*, as well as treatment with 3-oxo-C12-HSL barely affected PrrH expression (Figure 5B and S5A). However, *rhlI* deletion caused an increase in endogenous PrrH expression, which was attenuated by restoration of RhlI (Figure 5B) or C4-HSL (Figure S5B). More importantly, similar to overexpression of PrrH, deficiency in *rhlI* also led to downregulation of LasA, PhzC and PhzD but not of LasI, and restoration of RhlI rescued this phenomenon (Figure 5C).

Since the gene expression-regulated function of RhlI is mediated by the transcription factor (TF) RhlR, we further used the bioinformatics tool PRODORIC, a comprehensive database for gene regulation and expression in prokaryotes, to analyse the DNA motif recognized by RhlR. It seems that genes, such as *rhlAB*, *pa1L* and *migA*, with the specific DNA sequence 5’-CT(N12)AG-3’ or 5’-CT(N12)GC-3’ in the promoter region may be potential targets of RhlR. Based on this model, within the −100 bp region upstream of PrrH, two RhlR consensus binding sites were found (Figure 5D). Then, a DNA fragment of *prrH* promoter carrying these predicted sites was cloned upstream of the β-galactosidase gene in the pQF50-promoter reporter. When transformed into BW25113 (an *E. coli* strain without endogenous β-galactosidase activity), the β-galactosidase activity of pQF50-P*prrH* was greatly elevated compared to that of the control, while overexpression of RhlR impaired this activity (Figure 5E). Moreover, overexpression of RhlR also resulted in downregulation of PrrH (Figure 5F). These results suggest that the virulence regulatory role of PrrH is under the control of *rhl* system.

**Figure 6.**
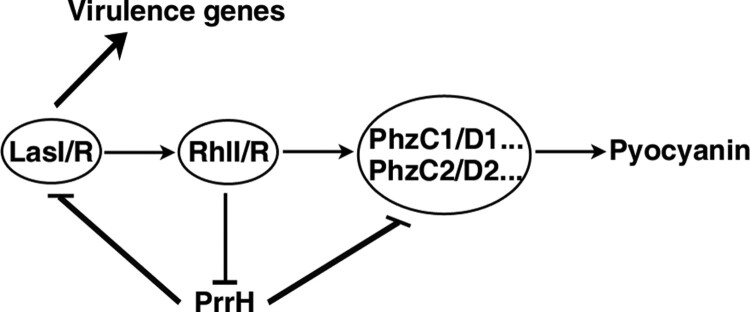
Schematic overview of the RhlI/R-PrrH-LasI/PhzC/PhzD signalling cascade and its implication in *P. aeruginosa* virulence.

Taken together, our results elucidate a novel RhlI/R-PrrH-LasI/PhzC/PhzD signalling cascade and further identify the importance of PrrH in inhibiting pyocyanin synthesis and virulence (Figure 6), which substantially extend our understanding of the *P. aeruginosa* QS circuit and the function of PrrH.

## Discussion

In this study, we disclose a novel QS/PrrH-PhzC/PhzD regulatory circuitry, that is, RhlI/R represses the expression of PrrH, whereas the expression of PrrH reduces the levels of LasI, RhlI and PhzC/PhzD, thereby inhibits QS signalling and abrogates QS-promoted virulence in *P. aeruginosa*. Our results elucidate the new regulation mechanism and function of PrrH.

One of the most fascinating aspects of the *prrF* locus sRNAs in *P. aeruginosa* is the tandem arrangement of the *prrF1* and *prrF2* genes, allowing for expression of a third sRNA, PrrH [27]. Most recent publications about *prrF* locus sRNAs have focused on the role of PrrF in iron homeostasis or PQS regulation [17,19,27,28]. Few reports suggest that there is an extra haem-responsive function for PrrH, as a result of the unique sequence derived from the *prrF1*-*prrF2* intergenic sequence (PrrH_IG_) [20]. In this study, we showed that deletion or overexpression of the whole sequence of *prrH* resulted in a change in virulence and bloodstream infection ability. However, our genetic manipulation not only changed the PrrH level but also caused gain or loss of PrrF expression. In addition, the recognition site of PrrH on LasI, PhzC and PhzD was not in PrrH_IG_ but shared with PrrF. Thus, the assessed function of PrrH in our work relies on that of the *prrH* locus sRNAs and not on PrrH only.

In *P. aeruginosa* PAO1, pyocyanin is synthesized from chorismate in a complex series of intermediates by enzymes encoded by the homologous *phzA1B1C1D1E1F1G1* and *phzA2B2C2D2E2F2G2* operons. The two *phz* operons are 98.3% identical at the DNA level [26,29]. As a result, the recognition site of PrrH in PhzC1/D1 is the same as that in PhzC2/D2. We confirmed that both PhzC1/D1 and PhzC2/D2 were targets of PrrH. More interestingly, the pairing bases among these four target mRNAs were conserved, meaning that the PrrH family used the same base-pairing site to recognize a protein family. In the perspective of evolution, this conservation means that these base-pairing events are likely to be physiologically relevant.

It has been shown that the transcription of quite a few sRNAs is regulated by TFs and that many sRNAs regulate TF-encoding genes. This strategy implies that TFs, sRNAs and target genes may form a complex regulatory circuit [8,9,30]. For example, in *Vibrio harveyi* and *Vibrio cholera*, LuxO activates the expression of the Qrr sRNAs, which in turn repress the expression of the QS master regulators LuxR and LuxO [30–33]; thus, sRNAs may function at the centre of QS circuits. *P. aeruginosa* is a model for studies of QS. However, the homologous Qrr sRNAs in *P. aeruginosa* have not yet been characterized. Here, we showed that RhlI/R directly repressed PrrH and promoted PhzC/D expression, while PhzC/D was negatively regulated by PrrH. This finding suggests that RhlI/R, PrrH and PhzC/D combine in a feed-forward loop to regulate pyocyanin production. On the other hand, PrrH also repressed RhlI/R expression (Figure S6) through targeting LasI, forming a negative-negative feedback loop to fine-tune QS. Our data suggest that *prrH* locus sRNAs may be the Qrr sRNAs of *P. aeruginosa*.

A few publications have reported several genes involved in the link between iron and QS systems in *P. aeruginosa*. MvfR, for example, was transcriptionally activated under low-iron concentrations or by the cell–cell signalling molecules 4-hydroxy-2-heptylquinoline (HHQ) and 3,4-dihydroxy-2-heptylquinoline (PQS), subsequently controlling transcription of iron-related genes and many virulence-related factors [34,35]. Previous works have disclosed that PrrF was transcriptionally repressed by Fur [19]. Its target mRNA involved in iron homeostasis was PhuS, and AntR and VreR were targets involved in virulence [17,27]. In our work, LasI and PhzC/D were identified as new virulence-associated targets for this locus sRNA. More importantly, we also revealed that the expression of PrrH was under the control of RhlI/R. To our knowledge, this study is the first time that a sRNA has been shown to connect AHL QS to iron homeostasis in *P. aeruginosa*.

In summary, we disclose a novel QS-PrrH regulatory circuit and demonstrate that PrrH is capable of repressing *P. aeruginosa* virulence and bloodstream infection ability *in vitro*. Our findings advance the fundamental understanding of QS circuits in *P. aeruginosa* and indicate that PrrH might be a potential target for therapeutic development.

### Experimental procedures

#### Bacterial strains, plasmids and growth conditions

The bacterial strains and plasmids used in this study are listed in Table S1. Bacteria were grown in LB medium or on LB plates containing 1.5% agar, unless otherwise indicated. When necessary, antibiotics were used at the following concentrations: 100 μg/ml ampicillin (AMP), 25 μg/ml gentamicin (GM), 16 μg/ml chloramphenicol (CM), and 2, 8, and 32 μg/ml AZM.

#### Plasmid construction

The whole sequence of PrrH or the coding sequences of LasI, RhlI and RhlR were cloned into the EcoRΙ site of pROp200 with a Ready-to-Use Seamless Cloning Kit (BBI, USA, Code NO. B632219) to generate expression vectors named pROp200-*prrH*, pROp200-*lasI*, pROp200-*rhlI* and pROp200-*rhlR*, respectively. For another PrrH expression vector, pSTV28-*prrH*, the *prrH* gene was cloned into the *Pst*I/*Hind* III sites of pSTV28.

To construct the target-*gfp* translational fusion vectors pUCP30T-*lasI*-*gfp,* pUCP30T-*phzC*-*gfp* and pUCP30T-*phzD*-*gfp*, the coding sequence of GFP was first cloned into *Xba*I/*Hind*III sites downstream of the P*_lac_* promoter in pUCP30T to generate the reporter vector pUCP30T-*gfp*, and then a wild-type fragment of LasI, PhzC or PhzD mRNA that contained putative binding sites for PrrH was PCR amplified and inserted into the *Xba*I/*Nco*I sites upstream of the first codon of GFP in the pUCP30T-*gfp* vector. pUCP30T-mRNA-*mut-gfp*, which carried a mutated sequence in the complementary site for PrrH was generated using fusion PCR based on wild-type pUCP30T-mRNA*-gfp*.

The plasmid pQF50 containing a promoter-less *lacZ* reporter was used for *prrH* promoter analysis. A DNA fragment of the *prrH* promoter spanning −92 to + 74 was PCR-amplified and inserted into *BamH*I/*Hind* III sites upstream of the *lacZ* reporter in pQF50 to generate pQF50-P*prrH*.

All constructs were confirmed by direct sequencing. Primers used in this study are listed in Table S2.

#### Construction of PAO1 prrH-deficient mutants

The sacB-based suicide vector system was employed for the knockout of *prrH* in PAO1 as described by Zeng et al in our previous work [21]. Briefly, the upstream and downstream recombinant fragment of *prrH* was cloned into *Xba*I/*Sac*I sites in pGSM to generate the recombinant plasmid pGSM-*ΔprrH*, and then it was transformed into PAO1 to generate a *prrH* mutant strain (PAO1Δ*prrH*).

#### Real-time PCR

Total RNA was extracted using RNAiso Plus reagent (Takara, Dalian, Liaoning, China). Reverse transcription (1 μg of total RNA) was performed with the PrimeScript RT Reagent Kit (Takara, Dalian, Liaoning, China; code No. RR047A). The cDNA was subjected to qPCR on a ViiA^TM^ 7 Dx system (Applied Biosystems, Foster, CA, USA) using SYBR Green qPCR Master Mix (Takara, Dalian, Liaoning, China). The expression levels of the target genes were normalized to the expression of an internal control gene (*rpoD*) using the 2^−ΔΔCt^ method. The sequences of the primers are listed in Table S2.

#### Fluorescence intensity measurement

*E. coli* DH5α carrying the reporter pUCP30T-mRNA-*gfp* or pUCP30T-mRNA-*mut-gfp* combined with pSTV28 or pSTV28-*prrH* was grown overnight (8∼10 h) at 37°C and was re-incubated to 0.5 McFarland standard (MCF), and then 100 μl of cultures were added into 3 mL of LB to grow for 6 h. Subsequently, cultures were collected by centrifugation and washed twice and resuspended in 0.9% NaCl, and 200 μl aliquots were transferred to black polystyrene 96-well microplates with a clear, flat bottom. The absorbance (Abs595) and fluorescence intensity (F485/535) were measured in a Tecan Infinity M1000Pro Reader. GFP activity was expressed in arbitrary units (AU) as F485/535/Abs595. All of the tests were carried out independently and at least in triplicate.

#### β-galactosidase assays

β-galactosidase assays were carried out by the Miller method when cells were grown to mid-log phase at 37°C [36]. All of the tests were carried out independently and at least in triplicate.

#### Pyocyanin production assay

Pyocyanin in 5 ml of *P. aeruginosa* culture supernatant was extracted with 3 ml of chloroform and 1 ml of 0.2 N HCl, and then the absorbance of the extract was measured at 520 and 600 nm. The concentration of pyocyanin was determined using the following formula: (A520/A600×17.072) = μg/ml [37]. All of the tests were performed independently and at least in triplicate.

#### Elastase assay

Elastase activity in *P. aeruginosa* cultures supernatant was determined by elastin-Congo red (ECR) (Sigma Chemical, America) assay [38]. Mid-exponential phase bacteria were inoculated in 3 ml of LB at an OD600 of 0.05 and grown for 8 h at 37°C. Then,200 μl culture supernatants were added to 800 μl ECR solution containing 0.1 M Tris (pH 7.2), 1 mM CaCl_2_ and 3 mg /ml ECR. Reaction tube was incubated 4 h at 37°C with shaking and then 100 μl of 0.12 M EDTA was added to stop reaction. Insoluble ECR was removed by centrifugation, and the OD495 was measured. All results were analyzed of three independent experiments.

#### Rhamnolipid assay

The detection of rhamnolipid was based on the experimental method used by Neissa M. Pinzon with some modifications [39]. Mid-exponential phase bacteria were resuspended in 3 ml of M9 minimal salts medium that contained 0.4% glucose, 2 mM MgSO4 and 100 µM CaCl_2_ at OD600 of 0.05 and incubated at 37°C for 8 h. 1ml of culture supernatant was acidified (pH 2.5 ± 0.2) using 1 N HCl, rhamnolipid was extracted with 4 ml chloroform. Then 3 ml of chloroform extract was allowed to react with 100 µl of 1 g/l of methylene blue and 2 ml of distilled water, the OD638 was measured. All results were analyzed of three independent experiments.

#### Biofilm formation assay

A biofilm formation assay was performed as described previously by Sara Carloni [13]. Mid-exponential phase bacteria were inoculated in 2 ml of LB at an OD600 of 0.05 in 12-well plates and grown for 24 h at 37°C. Biofilm cells attached to plates were stained with 1% crystal violet. Then, 2 ml of 95% ethanol was used to solubilize crystal violet-stained biofilm cells and measured at OD600. All analyses included three independent experiments.

#### Motility assays

*P. aeruginosa* strains were grown in LB medium to the mid-exponential phase, and 5 μl of the culture was spotted onto swarming medium (LB containing 0.5% agar and 5 g/l glucose) and swimming medium (tryptone broth containing 0.3% agar), respectively [40]. Swarming and swimming motility were observed after 16 h of incubation at 37°C. At least three replicates were measured for each sample.

#### Whole blood killing assay

Whole blood was partitioned into wells of a 96-well round bottom plate, and 1 × 10^6^ CFU of the indicated PAO1 strain in 0.9% NaCl was added to whole blood to produce a final concentration of 90% whole blood in a 200 μl volume. Samples were taken at time 0, 30, 60, 90, and 120 min, serially diluted, and plated on LB agar plates for CFU enumeration [41]. Experiments were performed at least in triplicate.

#### Ethics approval and consent to participate

For the whole blood killing assay, informed consent was obtained from each volunteer, and the study was approved by the Ethics Committee of Guangdong Provincial Hospital of Traditional Chinese Medicine (approval notice number: BE-2018-166-01).

#### Statistical analysis

Data of the results from multiple independent experiments are expressed as the mean ± standard deviation (SD). The differences between groups were analysed using Student’s t-test when two groups were compared or one-way ANOVA when more than two groups were compared. All analyses were performed using GraphPad Prism, version 5 (GraphPad Software, Inc., San Diego, CA, USA). Differences with a *P*-value (*P*) of 0.01 < *P* < 0.05 are represented by *, *P* < 0.01 are represented by **, and *P* < 0.001 are represented by ***.

## Supplementary Material

Supplemental Material
