# Supplementary material for: Identification of a novel RhlI/R-PrrH-LasI/Phzc/PhzD signalling cascade and its implication in P. aeruginosa virulence
Source: Emerg Microbes Infect. 2019 Nov 12;8(1):1658–67. doi: 10.1080/22221751.2019.1687262 (PMC6853234; doi:10.1080/22221751.2019.1687262)
Supplement: Supplemental Material [file TEMI_A_1687262_SM1602.zip › Table_S1._Strains_and_plasmids_used_in_the_study_final.docx]

**Table S1. Bacterial strains and plasmids.**

| **Strains** | **Genotype or characteristics** | **Source** |
| --- | --- | --- |
| ***Pseudomonas aeruginosa*** | | |
| PAO1 | wild-type strain，Gm^S^，Amp^R^ | Our lab |
| PAO1 *ΔlasI* | deficiency of *lasI*，Gm^S^，Amp^R^ | Our lab (1) |
| PAO1 *ΔrhlI* | deficiency of *rhlI*，Gm^S^，Amp^R^ | Our lab |
| PAO1 *ΔprrH* | deficiency of *prrH*，Gm^S^，Amp^R^ | This work |
| ***Escherichia coli*** | | |
| DH5α | wild-type strain，Gm^S^，Amp^S^ | Our lab |
| BW25113 | wild-type strain，Gm^S^，Amp^S^ | Our lab |
| ***Plasmids*** | | |
| pQF50 | Promter-less *la*cZ reporter plasmid，Amp^R^ | Our lab |
| pQF50-P*prrH* | pQF50 derivative, *la*cZ reporter plasmid，controlled by the constitutive *prrH* promoter，Amp^R^ | This work |
| pROp200 | Control plasmid based on pBBR1 MCS-5，Gm^R^ | (2) |
| pROp200-*lasI* | pROp200 derivative，*P. aeruginosa* PAO1 *lasI* overexpression plasmid，controlled by the constitutive P*_tac_* promoter，Gm^R^ | This work |
| pROp200-*rhlI* | pROp200 derivative，*P. aeruginosa* PAO1 *rhlI* overexpression plasmid，controlled by the constitutive P*_tac_* promoter，Gm^R^ | This work |
| pROp200-*rhlR* | pROp200 derivative，*P. aeruginosa* PAO1 *rhlR* overexpression plasmid，controlled by the constitutive P*_tac_* promoter，Gm^R^ | This work |
| pROp200-*prrH* | pROp200 derivative，*P. aeruginosa* PAO1 *prrH* overexpression plasmid，controlled by the constitutive P*_tac_* promoter，Gm^R^ | This work |
| pSTV28 | Control plasmid ；containing P*_lac_* promoter，Cm^R^ | Our lab |
| pSTV28-*prrH* | pSTV28 derivative，*P. aeruginosa* PAO1 *prrH* overexpression plasmid，controlled by the constitutive P*_lac_* promoter，Cm^R^ | This work |
| pUCP24T | 370bp oriT fragment from pCVD442 cloned into pUCP24, ori1600, Gm^R^ | Our lab |
| pUCP30T | Pucp24T derivative，P*_lac_* promoter，Gm^R^ | Our lab |
| pUCP30T-*gfp* | pUCP30T and pET28-*gfp* derivative；Control plasmid；*gfp* reporter plasmid；*gfp* controlled by the constitutive P*_lac_* promoter，Gm^R^ | This work |
| pUCP30T-*lasI-gfp* | pUCP30T-*gfp* derivative；containing transcription fusion of *lasI-gfp*； *las-gfp* controlled by the constitutive P*_lac_* promoter，Gm^R^ | This work |
| pUCP30T-*phzC*-*gfp* | pUCP30T-gfp derivative；containing transcription fusion of *phzC*-*gfp*；*phzC*-*gfp* controlled by the constitutive P*_lac_* promoter，Gm^R^ | This work |
| pUCP30T-*phzD-gfp* | pUCP30T-*gfp* derivative；containing transcription fusion of *phzD*-*gfp*；*phzD*-*gfp* controlled by the constitutive P*_lac_* promoter，Gm^R^ | This work |
| pUCP30T-*lasI-mut-gfp* | pUCP30T-*gfp* derivative; *lasI* with mutated in the PrrH sRNA interaction zone; *lasI-mut-gfp* controlled by the constitutive P*_lac_* promoter，Gm^R^ | This work |
| pUCP30T-*phzC-mut-gfp* | pUCP30T-*gfp* derivative; *phzC* with mutated in the PrrH sRNA interaction zone; *phzC-mut-gfp* controlled by the constitutive P*_lac_* promoter，Gm^R^ | This work |
| pUCP30T-*phzD-mut-gfp* | pUCP30T-*gfp* derivative; *phzD* with mutated in the PrrH sRNA interaction zone; *phzD-mut-gfp* controlled by the constitutive P*_lac_* promoter，Gm^R^ | This work |

Cm^R^, Gm^R^ and Amp^R^ stand for chloramphenicol, gentamycin and ampicillin resistance, respectively.

**References:**

1. Zeng, J., Zhang, N., Huang, B., Cai, R., Wu, B., E, S., Fang, C. and Chen, C. (2016) Mechanism of azithromycin inhibition of HSL synthesis in Pseudomonas aeruginosa. *SCI REP-UK*, 6.

2. Lu, P., Wang, Y., Zhang, Y., Hu, Y., Thompson, K.M. and Chen, S. (2016) RpoS-dependent sRNA RgsA regulates Fis and AcpP inPseudomonas aeruginosa. *MOL MICROBIOL*, 102, 244-259.
