# Supplementary material for: Identification of a novel RhlI/R-PrrH-LasI/Phzc/PhzD signalling cascade and its implication in P. aeruginosa virulence
Source: Emerg Microbes Infect. 2019 Nov 12;8(1):1658–67. doi: 10.1080/22221751.2019.1687262 (PMC6853234; doi:10.1080/22221751.2019.1687262)
Supplement: Supplemental Material [file TEMI_A_1687262_SM1602.zip › Table_S2._Oligonucleotides_used_in_this_study._final.docx]

**Table S2. Sequences of RNA and DNA oligonucleotides**

| Number | Primers | Primer sequence^*^ (5’→3’) | Use for |
| --- | --- | --- | --- |
| **Primers for gene cloning** | | | |
| 1 | In28-*prrH*-F(P) | TCCTCTAGAGTCGACCTGCAGCTGCTTAACCGGGAAGTGAC | pSTV28-*prrH* |
| 2 | In28-*prrH*-R(H) | ACGACGGCCAGTGCCAAGCTTAAGAAGGCACGTTGAAGGAA |  |
| 3 | In200-*prrH*-F(E) | ATCGGCTCGTATAATGAATTCCTGCTTAACCGGGAAGTGAC | pROp200-*prrH* |
| 4 | In200-*prrH*-R(E) | CGAATTTTAACAAAAGAATTCAAGAAGGCACGTTGAAGGAA |  |
| 5 | In200-*lasI*-F(E) | ATCGGCTCGTATAATGAATTCTCCGGGTTCACCGAAATC | pROp200-*lasI* |
| 6 | In200-*lasI*-R(E) | CGAATTTTAACAAAAGAATTCTCATGAAACCGCCAGTCG |  |
| 7 | In200-*rhlI*-F(E) | ATCGGCTCGTATAATGAATTCATGATCGAATTGCTCTCT | pROp200-*rhlI* |
| 8 | In200-*rhlI*-R(E) | CGAATTTTAACAAAAGAATTCTCACACCGCCATCGACAG |  |
| 9 | In200-*rhlR*-F(E) | ATCGGCTCGTATAATGAATTCATGAGGAATGACGGAGGCTT | pROp200-*rhlR* |
| 10 | In200-*rhlR*-R(E) | CGAATTTTAACAAAAGAATTCTCAGATGAGACCCAGCGCCG |  |
| 11 | P*prrH*-F(B) | CGGGATCCCTGAGGCCCATTCCAGAG | pQF50-P*_prrH_* |
| 12 | P*prrH*-R(H) | CCCAAGCTTGCCTGATGAGGAGATAATCTGA |  |
| **Primers for target-*gfp* translational fusion vectors** | | | |
| 13 | 30T-*lasI*-F | AGTTCTAGACGGGTTCACCGAAATCTATC | pUCP30T-*lasI*-*gfp* |
| 14 | 30T-*lasI*-R | AGTCCATGGCATCGATTTCCATCTCGTC |  |
| 15 | 30T-*phzC*-F | GCTCTAGAATGCTCGACTACGAGCTGTCGATG | pUCP30T-*phzC*-*gfp* |
| 16 | 30T-*phzC*-R | CATGCCATGGGGTCGCTCAGCCAGATCAC |  |
| 17 | 30T-*phzD*-F | GCTCTAGAATGGACTGGCTGCTGACCAAGTG | pUCP30T-*phzC*-*gfp* |
| 18 | 30T-*phzD*-R | CATGCCATGGGCTGGATGTCGTTGGAGTAG |  |
| 19 | 30T-*lasI*-RM | AGCAGTTTTTTAAGCTTGAGATCGCGCCGACCAAT | pUCP30T-*lasI-mut*-*gfp* |
| 20 | 30T-*lasI*-FM | ATTGGTCGGCGCGATCTCAAGCTTAAAAAACTGCT |  |
| 21 | 30T-*phzC*-RM | GAGCGCCAGCATCACCTTACGGCCGATCTC | pUCP30T-*phzC-mut*-*gfp* |
| 22 | 30T-*phzC*-FM | GAGATCGGCCGTAAGGTGATGCTGGCGCTC |  |
| 23 | 30T-*phzD*-RM | GCACAGCACCATCACCTTACGGCCGGCGGC | pUCP30T-*phzD-mut*-*gfp* |
| 24 | 30T-*phzD*-FM | GCCGCCGGCCGTAAGGTGATGGTGCTGTGC |  |
| **Primers for qPCR** | | | |
| 25 | RT-*rpoD*-F | CTGAAGATCGCCAAAGAGCC | qRT-PCR |
| 26 | RT-*rpoD*-R | GTGTGGTCGGTGTTCATGTC |  |
| 27 | RT-*prrH*-F | CGAGATCAGCCGGTAAGC | qRT-PCR |
| 28 | RT-*prrH*-R | CAAGCCGGTTCTCATTCATT |  |
| 29 | RT-*lasI*-F | CGTGCTCAAGTGTTCAAGGA | qRT-PCR |
| 30 | RT-*lasI*-R | AAAACCTGGGCTTCAGGAGT |  |
| 31 | RT-*lasA*-F | ACCAGATCCAGGTGAGCAAC | qRT-PCR |
| 32 | RT-*lasA*-R | CGTTGTCGTAGTTGCTGGTG |  |
| 33 | RT-*rhlI*-F | CTACCGGCATCAGGTCTTCA | qRT-PCR |
| 34 | RT-*rhlI*-R | GTTTCGCTGCACAGGTAGG |  |
| 35 | RT-*rhlA*-F | AGCTGGGACGAATACACCAC | qRT-PCR |
| 36 | RT-*rhlA*-R | GACTCCAGGTCGAGGAAATG |  |
| 37 | RT-*rhlR*-F | GTTGCATGATCGAGTTGCTG | qRT-PCR |
| 38 | RT-*rhlR*-R | TGGATGTTCTTGTGGTGGAA |  |
| 39 | RT-*phzC*-F | CGGATCCTCAAGGGCTATG | qRT-PCR |
| 40 | RT-*phzC*-R | GTGGGTCGAACCGAGATAGA |  |
| 41 | RT-*phzD*-F | GACATGCAGCGCTACTTCCT | qRT-PCR |
| 42 | RT-*phzD*-R | CCCAGAAGTCCTTGAGCAGA |  |

^*^ The underlined sequences denote restriction enzyme site.
