# Supplementary figures and images for: Identification of a novel RhlI/R-PrrH-LasI/Phzc/PhzD signalling cascade and its implication in P. aeruginosa virulence
Source: Emerg Microbes Infect. 2019 Nov 12;8(1):1658–67. doi: 10.1080/22221751.2019.1687262 (PMC6853234; doi:10.1080/22221751.2019.1687262)

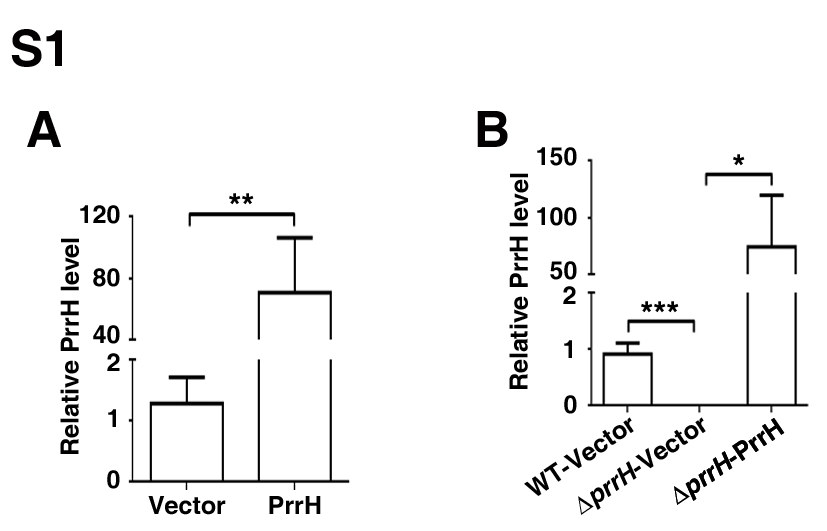

Supplement: Supplemental Material [file TEMI_A_1687262_SM1602.zip › Sup.Fig.1_final.tif]

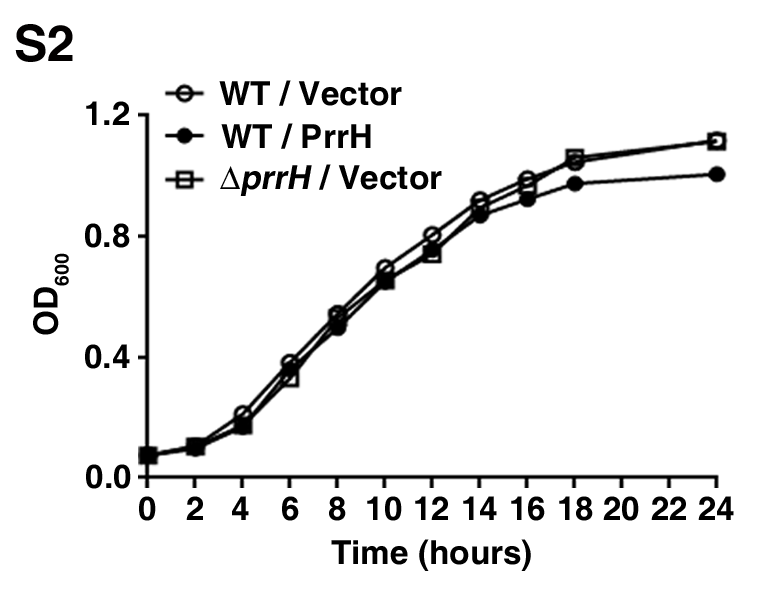

Supplement: Supplemental Material [file TEMI_A_1687262_SM1602.zip › Sup.Fig.2_final.tif]

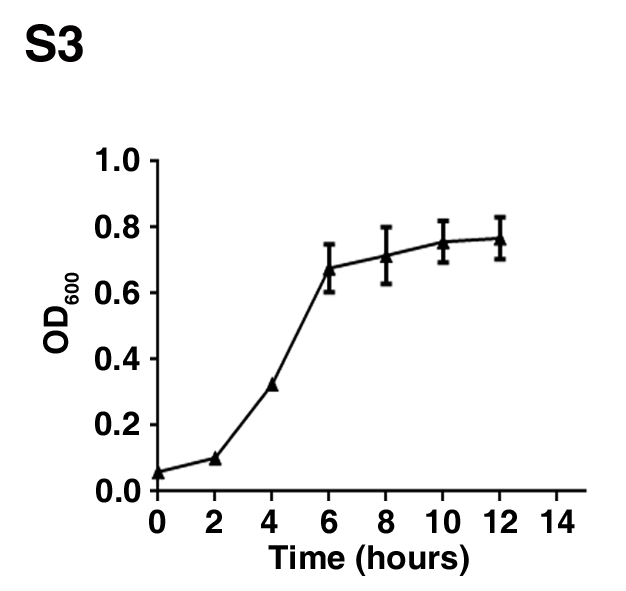

Supplement: Supplemental Material [file TEMI_A_1687262_SM1602.zip › Sup.Fig.3_final.tif]

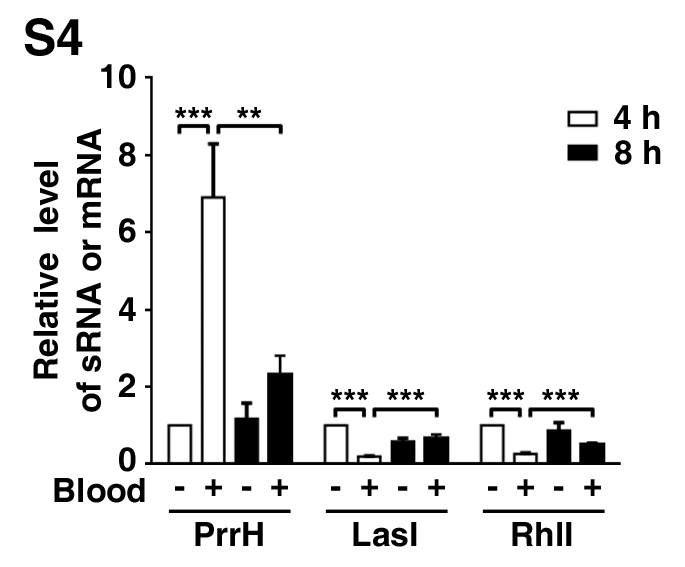

Supplement: Supplemental Material [file TEMI_A_1687262_SM1602.zip › Sup.Fig.4._final.tif]

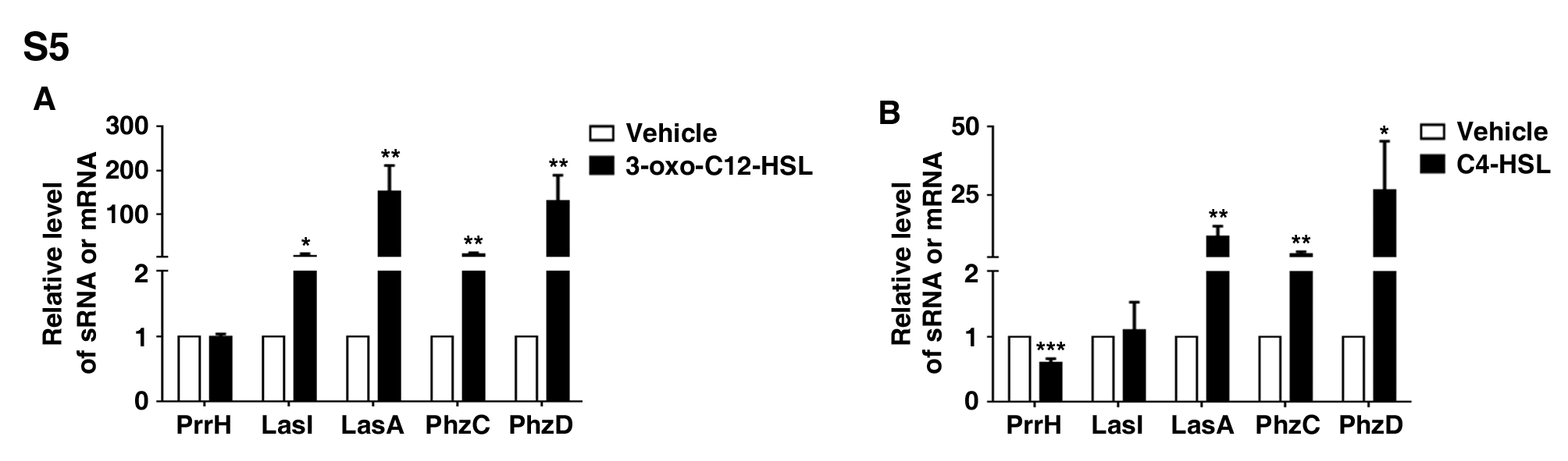

Supplement: Supplemental Material [file TEMI_A_1687262_SM1602.zip › Sup.Fig.5_final.tif]

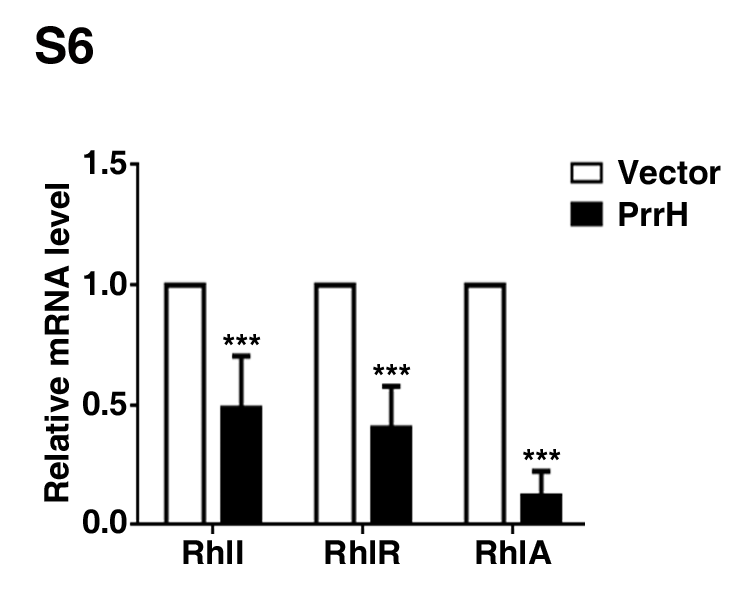

Supplement: Supplemental Material [file TEMI_A_1687262_SM1602.zip › Sup.Fig.6_final.tif]
